# Impact of an amino acid deletion detected in the hemagglutinin (HA) antigenic site of swine influenza A virus field strains on HA antigenicity

**DOI:** 10.1128/jvi.01820-25

**Published:** 2026-02-19

**Authors:** Taichi Nakano, Mana Esaki, Akiha Inoue, Fumiko Koike, Kosuke Okuya, Makoto Ozawa

**Affiliations:** 1 Joint Faculty of Veterinary Medicine, Kagoshima University 199253 , Kagoshima, Japan; 2 Joint Graduate School of Veterinary Medicine, Kagoshima University 12851, Kagoshima, Japan; 3 Swine Management Consultation K.K., Atsugi, Japan; University Medical Center Freiburg, Freiburg, Germany

**Keywords:** antigenicity, hemagglutinin, swine influenza

## Abstract

Swine influenza A virus (swIAV) is an important pathogen with regard to both the swine industry and public health. The pandemic A(H1N1) 2009 outbreak was caused by the swine-origin pandemic A(H1N1) 2009 [A(H1N1)pdm09] virus. Several reports have shown that several amino acid substitutions in the hemagglutinin (HA) antigenic sites can alter HA antigenicity. However, the impact of the amino acid deletion at position 155 on HA antigenicity remains unknown. In this study, we have isolated 11 samples of swIAVs from seven pig farms in Japan and found an amino acid deletion at position 155 of the HA region in one of the isolates of the H1N2 subtype. To examine the impact of this amino acid deletion on viral replication and HA antigenicity, we generated recombinant influenza A viruses possessing the H1 HA gene encoding either an artificial insertion or deletion of glycine at position 155. The growth kinetics of these recombinant viruses in two different cell lines demonstrated that the effect of amino acid deletion at position 155 of H1 HA on viral replication is limited. In contrast, microneutralization assay-based neutralization titers revealed that amino acid deletion significantly altered HA antigenicity. These results demonstrate that a naturally occurring amino acid deletion at position 155 in an H1 HA antigenic site can markedly alter HA antigenicity with only a limited impact on replication *in vitro*, highlighting the need to monitor such variants in swine populations and to assess their zoonotic potential.

**IMPORTANCE:**

Influenza A viruses circulating in pigs are of major concern because they can reduce herd productivity and sometimes infect humans. Understanding how these viruses change their surface proteins is essential for predicting their evolution and preventing outbreaks. This study focuses on swine influenza A virus isolated from pig farms in Japan and investigates the effect of a specific amino acid deletion in the hemagglutinin protein, which plays a key role in viral recognition by the host immune system. The results show that this deletion does not greatly affect virus growth but markedly alters how the immune system recognizes the virus. Such antigenic changes may allow the virus to evade existing immunity in pig populations and potentially increase the risk of transmission to humans. These findings underscore the need for continued surveillance of such variants in pigs and for future studies to evaluate their possible zoonotic implications.

## INTRODUCTION

Swine influenza A virus (swIAV), influenza A virus isolated from pigs, causes respiratory symptoms in pigs (1). SwIAV is highly contagious and circulating in pig populations globally (2–4). The economic loss to the swine industry due to swIAV infection is estimated to be approximately US$3 per head and can go up to US$10 when coinfected with other pathogens, such as porcine reproductive and respiratory syndrome virus (3, 5). Furthermore, several zoonotic transmissions of swIAVs to humans have been reported (6). More importantly, a reassortant virus of multiple swIAV strains that originally emerged in pigs infected with avian and human influenza viruses caused a pandemic in humans in 2009 (7–9). Therefore, swIAV is an important pathogen in regard to the swine industry as well as public health.

The swIAV RNA genome consists of eight gene segments (10), among which, the hemagglutinin (HA) gene segment encodes HA, a viral surface protein that is the primary target of the host antibody responses (11). In particular, five structural regions in HA of H1 subtype (H1 HA), that is, antigenic sites Sa, Sb, Ca1, Ca2, and Cb, have been identified as immunodominant antigenic regions (12–14). Therefore, the amino acid sequences at these antigenic sites are key determinants of HA antigenicity.

Since the outbreak of pandemic A(H1N1) 2009 caused by swine-origin pandemic A(H1N1) 2009 [A(H1N1)pdm09] viruses, the viruses circulating in humans have been reverse introduced in pig populations globally, followed by the generation and circulation of genetic reassortants between A(H1N1)pdm09 viruses and endemic classical H1N2 swIAVs on a regional basis (15, 16). Several reports have described that several amino acid substitutions found in the HA antigenic sites of viruses belonging to the A(H1N1)pdm09 lineage can alter their HA antigenicity (17–20). In particular, a single amino acid substitution at position 155 of H1 HA (H1 numbering was applied throughout the manuscript), which is one of the amino acids that compose the antigenic site Sa (12, 21–23), was demonstrated to significantly affect HA antigenicity (18–21, 24); however, the impact of deletion at this position on HA antigenicity remains unknown.

In this study, we isolated 11 swIAVs from seven pig farms in Japan, followed by their genetic characterization. We then found an amino acid deletion at position 155 of the H1 HA in 1 of the 11 swIAV isolates. Therefore, this study aimed to delineate the impact of amino acid deletion on viral replication and HA antigenicity using artificially generated recombinant influenza A viruses.

## RESULTS

### Genetic characterization of swIAV isolates

**Fig 1:**
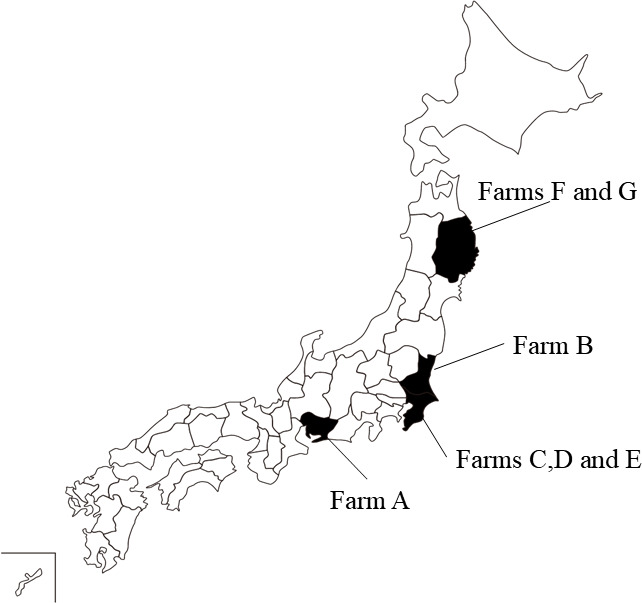
Geographical location of the farms. Prefectures where the farms were located are shown in black.

We isolated 11 swIAV strains from swine samples collected from seven pig farms in Japan: one isolate each from farms A to C and two isolates each from farms D to G (Fig. 1). The nucleotide sequences of the full-length HA and NA genes were determined to identify the HA and NA subtypes of the 11 swIAV isolates. The sequences revealed that our swIAVs were classified into two subtypes: H1N1 (two isolates) and H1N2 (nine isolates; Table 1). The nucleotide sequences of both genes from two isolates each from Ffarms D to G were almost identical.

**TABLE 1:** Swine influenza viruses analyzed in this study

Farm	Virus	Specimen source	Genes sequenced	Accession no.^*a*^
A	A/swine/Aichi/KU-FKAB/2018 (H1N2)	Lung tissue	Full genome	EPI_ISL_20155794
B	A/swine/Ibaraki/KU-FK11/2020 (H1N1)	Oral fluid	Full genome	EPI_ISL_20155795
C	A/swine/Chiba/KU-YH2/2018 (H1N1)	Nasal swab	Full genome	EPI_ISL_20155796
D	A/swine/Chiba/KU-YH5/2020 (H1N2)	Nasal swab	Full genome	EPI_ISL_20155797
	A/swine/Chiba/KU-YH9/2020 (H1N2)	Nasal swab	Full genome	EPI_ISL_20155798
E	A/swine/Chiba/KU-YH821-2/2021 (H1N2)	Nasal swab	Full genome	EPI_ISL_20155799
	A/swine/Chiba/KU-YH821-4/2021 (H1N2)	Nasal swab	HA and NA	EPI_ISL_20155800
F	A/swine/Iwate/KU-YH3-5/2021 (H1N2)	Nasal swab	Full genome	EPI_ISL_20155801
	A/swine/Iwate/KU-YH3-8/2021 (H1N2)	Nasal swab	HA and NA	EPI_ISL_20155802
G	A/swine/Iwate/KU-YH4-1/2021 (H1N2)	Nasal swab	Full genome	EPI_ISL_20155803
	A/swine/Iwate/KU-YH4-2/2021 (H1N2)	Nasal swab	HA and NA	EPI_ISL_20155804

^*a*^ All GISAID isolate information can be accessed by searching the corresponding accession numbers (e.g., EPI_ISL_XXXXXX) within the GISAID EpiFlu database (https://www.gisaid.org).

To further characterize the genetics of these 11 swIAV isolates, the nucleotide sequences of the remaining six genes from A/swine/Chiba/KU-YH5/2020 (H1N2) and A/swine/Chiba/KU-YH9/2020 (H1N2), both of which were isolated from Ffarm D, were determined. We demonstrated that the nucleotide sequences of all six internal genes from these two isolates were almost identical, indicating that isolates from the same pig farms were genetically equivalent. Therefore, we selected one isolate each from farms E to G, that is, A/swine/Chiba/KU-YH821-2/2021 (H1N2), A/swine/Iwate/KU-YH3-5/2021 (H1N2), and A/swine/Iwate/KU-YH 4-1/2021 (H1N2) from farms E, F, and G, respectively, for internal gene sequencing (Table 1).

**Fig 2:**
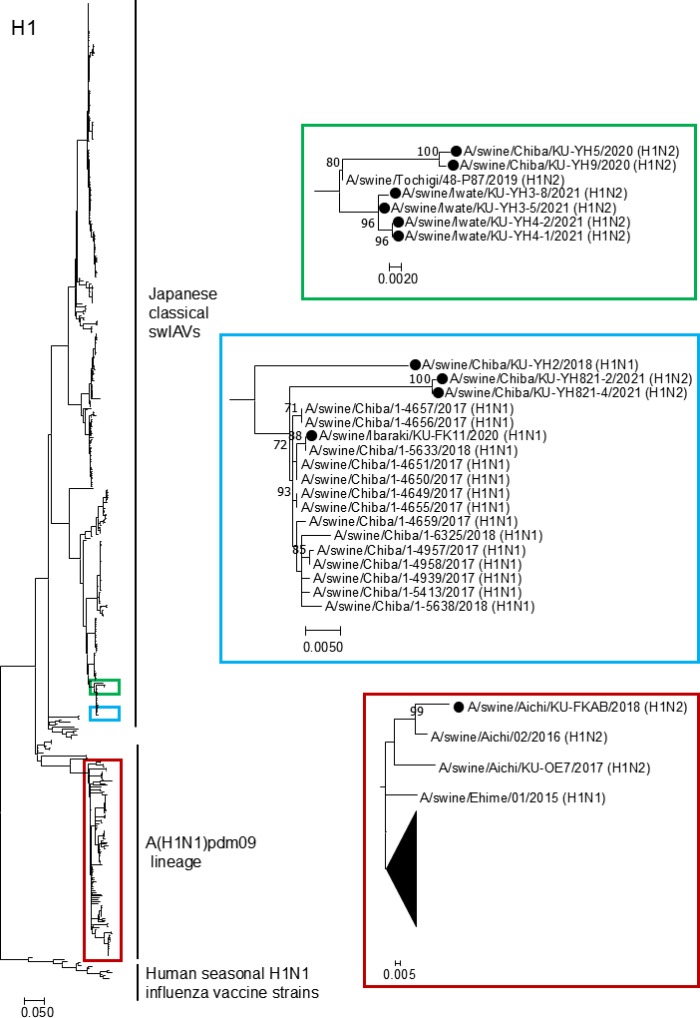
Phylogenetic tree of the H1 HA genes. The H1 HA gene nucleotide sequences from 11 swIAV isolates determined in this study were phylogenetically compared with those from Japanese swIAVs and human seasonal H1N1 influenza vaccine strains. The colored regions of the phylogenetic tree on the left side are shown in the enlarged views of the corresponding colors on the right side. To save space, the majority of A(H1N1)pdm09 viruses, including the human A(H1N1)pdm09 vaccine strains, collapse and form a triangle. H1 isolates are indicated by black circles. Bootstrap values of less than 70 are not shown.

To clarify the genetic relationship between our swIAV isolates and other swIAVs circulating in Japan and their human counterparts, the individual genes from our swIAV isolates were phylogenetically compared with those from Japanese swIAVs and human seasonal influenza vaccine strains. The phylogenetic tree of the H1 HA gene (Fig. 2) showed that the HA gene segment from one of our swIAV isolates, AB strain, belonged to the A(H1N1)pdm09 virus lineage, whereas those from the remaining 10 isolates were genetically close to those from the Japanese classical H1N2 swIAVs that have been circulating in Japan since the end of the 20th century at the latest.

**Fig 3:**
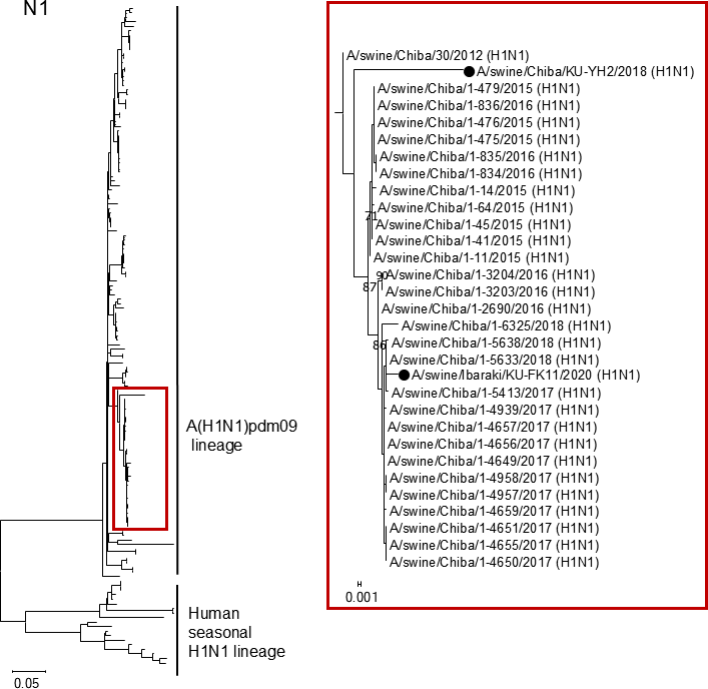
Phylogenetic trees of the N1 NA genes. The N1 NA gene nucleotide sequences from two swIAV isolates determined in this study were phylogenetically compared with those from Japanese swIAVs and human seasonal H1N1 influenza vaccine strains. The colored region of the phylogenetic tree on the left side is shown in the enlarged view of the corresponding colors on the right side. Our N1 isolates are indicated by black circles. Bootstrap values of less than 70 are not shown.

**Fig 4:**
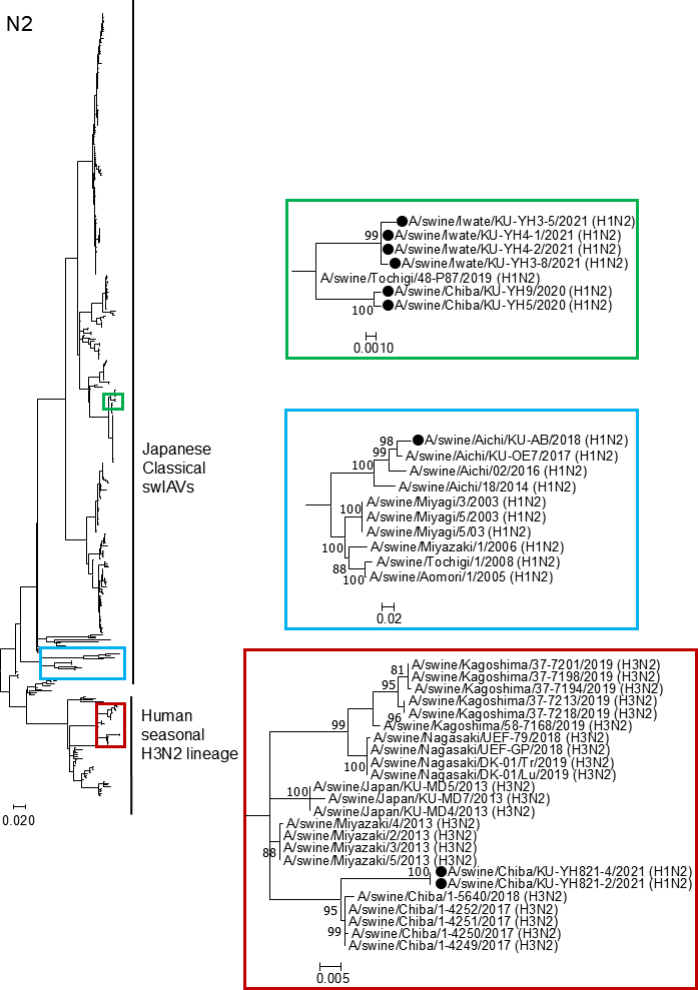
Phylogenetic trees of the N2 NA genes. The N2 NA gene nucleotide sequences from nine swIAV isolates determined in this study were phylogenetically compared with those from Japanese swIAVs and human seasonal H3N2 influenza vaccine strains. The colored regions of the phylogenetic tree on the left side are shown in the enlarged views of the corresponding colors on the right side. Our N2 isolates are indicated by black circles. Bootstrap values of less than 70 are not shown.

The phylogenetic tree of the N1 NA gene (Fig. 3) showed that the NA gene segments from our two N1 isolates, A/swine/Chiba/KU-YH2/2018 (H1N1) and A/swine/Ibaraki/KU-FK11/2020 (H1N1), belonged to the A(H1N1)pdm09 virus lineage. The phylogenetic tree of the N2 NA gene (Fig. 4) demonstrated that the NA gene segments from two of our nine N2 isolates, A/swine/Chiba/KU-YH821-2/2021 (H1N2) and A/swine/Chiba/KU-YH821-4/2021 (H1N2), were classified as human seasonal H3N2 influenza virus lineages. The N2 NA genes from the remaining seven H1N2 isolates were genetically close to those from the Japanese classical H1N2 swIAVs (Fig. 4).

Most of the internal gene segments (PB2, PB1, PA, NP, MP, and NS) from our swIAV isolates belonged to the A(H1N1)pdm09 virus lineage (Table 2). However, the NP gene segments from two H1N1 isolates, A/swine/Chiba/KU-YH2/2018 (H1N1) and A/swine/Ibaraki/KU-FK11/2020 (H1N1), were derived from Japanese classical H1N2 swIAVs, although their HA and NA gene segments were phylogenetically distant from each other (Fig. 2 and 4). These results indicate variations in the genetic constellation of swIAVs circulating in Japanese pig populations, even for the same subtype.

**TABLE 2:** Genetic origins of swine influenza viruses used in this study

Farm	Virus	HA	NA	PB2	PB1	PA	NP	M	NS
A	A/swine/Aichi/KU-FKAB/2018 (H1N2)	pdm09^*a*^	Classical	pdm09	pdm09	pdm09	pdm09	pdm09	pdm09
B	A/swine/Chiba/KU-YH2/2018 (H1N1)	Classical^*b*^	pdm09	pdm09	pdm09	pdm09	Classical	pdm09	pdm09
C	A/swine/Ibaraki/KU-FK11/2020 (H1N1)	Classical	pdm09	pdm09	pdm09	pdm09	Classical	pdm09	pdm09
D	A/swine/Chiba/KU-YH5/2020 (H1N2)	Classical	Classical	pdm09	pdm09	pdm09	pdm09	pdm09	pdm09
	A/swine/Chiba/KU-YH9/2020 (H1N2)	Classical	Classical	pdm09	pdm09	pdm09	pdm09	pdm09	pdm09
E	A/swine/Iwate/KU-YH3-5/2021 (H1N2)	Classical	Classical	pdm09	pdm09	pdm09	pdm09	pdm09	pdm09
	A/swine/Iwate/KU-YH3-8/2021 (H1N2)	Classical	Classical	–^*d*^	–	–	–	–	–
F	A/swine/Chiba/KU-YH821-2/2021 (H1N2)	Classical	Human^*c*^	pdm09	pdm09	pdm09	pdm09	pdm09	pdm09
	A/swine/Chiba/KU-YH821-4/2021 (H1N2)	Classical	Human	–	–	–	–	–	–
G	A/swine/Iwate/KU-YH4-1/2021 (H1N2)	Classical	Classical	pdm09	pdm09	pdm09	pdm09	pdm09	pdm09
	A/swine/Iwate/KU-YH4-2/2021 (H1N2)	Classical	Classical	–	–	–	–	–	–

^*a*^ pdm09: gene segments belonging to the A(H1N1)pdm09 virus lineage.

^*b*^ Classical: gene segments belonging to the Japanese classical H1N2 swIAVs.

^*c*^ Human: gene segments belonging to the human seasonal H3N2 influenza virus lineage.

^*d*^ –: not sequenced.

### Amino acid variation at position 155 of H1 HA

Intriguingly, the nucleotide sequence of the HA gene from the AB strain revealed an amino acid deletion at position 155 of the H1 HA. Previous studies have demonstrated that the amino acid at this position, which comprises the antigenic site Sa of HA (12, 21–23), significantly affects HA antigenicity (18, 21, 24).

To investigate amino acid variation at position 155 of the H1 HA, nucleotide sequence data for the HA genes from human, avian, and swine-origin influenza A viruses of the H1 subtype were retrieved from the Global Initiative on Sharing Avian Influenza Data (GISAID) database and aligned. Glycine was the most prevalent amino acid at the analyzed position: 94.2% in humans, 95.9% in avians, and 55.2% in swine viruses. More importantly, the proportion of amino acid deletions at the analyzed position was quite limited: 0% in humans, 0% in avians, and 0.17% (22 strains) in swine viruses. These results indicated that the amino acid at position 155 of the H1 HA was rarely deleted.

### Impact of the amino acid deletion at position 155 of H1 HA on viral replication

**Fig 5:**
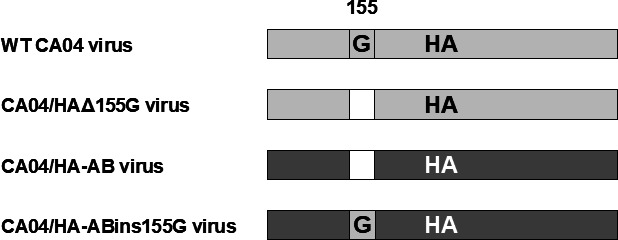
Schematic diagram of HA from four recombinant influenza A viruses generated in this study. HA from the WT CA04 virus (light gray bar) possesses glycine (G) at position 155. HA from the CA04/HAΔ155G virus was the CA04 strain-derived HA whose amino acid at position 155 was artificially deleted (shown by the white bar). The amino acid at position 155 in HA from the CA04/HA-AB virus (shown by a dark gray bar) was originally deleted. The HA from CA04/HA-ABins155G virus was an AB strain-derived HA with an artificial insertion G at amino acid position 155. Numbers above the bars indicate the amino acid positions of HA (H1 numbering).

To assess the effect of the amino acid deletion at position 155 of H1 HA on viral properties, we artificially generated four recombinant viruses based on an early A(H1N1)pdm09 isolate, CA04 strain, by reverse genetics. These four viruses possessed (Fig. 5) the H1 HA gene from the wild-type AB strain (CA04/HA-AB virus), AB strain-derived H1 HA gene with an artificial G insertion at position 155 (CA04/HA-ABins155G virus), H1 HA gene from the CA04 strain (WT CA04 virus), or CA04 strain-derived H1 HA gene with an artificial G deletion at position 155 (CA04/HAΔ155G virus).

**Fig 6:**
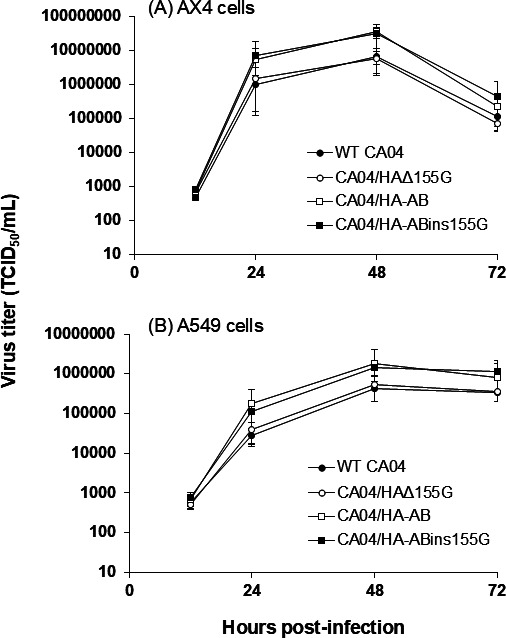
Growth kinetics of artificially generated four recombinant viruses. AX4 (**A**) and A549 (**B**) cells were inoculated with the WT CA04, CA04/HAΔ155G, CA04/HA-AB, and CA04/HAins155G viruses at a multiplicity of infection of 0.001. Supernatants collected at 12, 24, 48, and 72 hpi were analyzed for virus titers by 50% tissue culture infectious dose (TCID_50_) assays in AX4 cells. Error bars indicate standard deviations of triplicate experiments.

To assess the impact of amino acid deletion on viral replication, we first determined the *in vitro* growth kinetics of the four recombinant viruses in AX4 cells, which were previously demonstrated to be highly susceptible to swIAVs (25). No significant difference in growth kinetics was observed between CA04/HA-AB and CA04/HA-ABins155G viruses. Similarly, the growth kinetics of WT CA04 and CA04/HAΔ155G viruses were comparable (Fig. 6A). Although the peak titers of the WT CA04 virus in AX4 cells were modest compared with some previous reports, they were highly reproducible between independent experiments, and the relative growth characteristics among the recombinant viruses were consistent across replicates.

We next examined whether the amino acid deletion at position 155 influences viral replication in human respiratory epithelial A549 cells. Consistent with the results obtained in AX4 cells, no appreciable differences in growth kinetics were detected between CA04/HA-AB and CA04/HA-ABins155G viruses or between WT CA04 and CA04/HAΔ155G viruses in A549 cells (Fig. 6B). Together, these results indicated that the effect of amino acid deletion at position 155 of H1 HA on viral replication was limited.

### Impact of the amino acid deletion on HA antigenicity

To examine the impact of amino acid deletion at position 155 of H1 HA on HA antigenicity, the four recombinant viruses were antigenically characterized by microneutralization (MN) assays using 10 clones of monoclonal antibodies (mAbs) that recognize G at position 155 of H1 HA from A/Narita/1/2009 (H1N1), the first A(H1N1)pdm09 isolate in Japan (Table 3). The neutralization titers of each mAb against the WT CA04 virus were 4- to 500-fold higher than those against the CA04/HAΔ155G virus. In contrast, the neutralization titers of each mAb against the CA04/HA-AB virus were 4 to 160-fold lower than those against the CA04/HA-ABins155G virus.

**TABLE 3:** MN titer of four recombinant viruses artificially generated with the ferret antiserum and mAbs

Virus	MN titer^*a*^ with:										
	Ferret antiserum	mAb^*b*^									
		N230	N334	N73	N327	N329	N343	N345	N408	3N38	3N64
WT CA04	10,240	4,000	2,560	2,560	2,560	4,000	128,000	8,000	4,000	16,000	64
CA04/HAΔ155G	1,280	30	160	320	640	8	2,000–4,000	640	4	60	8
CA04/HA-ABins155G	160–320	40	128	80	160	32	32	160	40	320	4
CA04/HA-AB	<20	< 1	32	<1	20	2	2	<1	10	20	<1

^*a*^ The MN titer is expressed as the reciprocal of the highest antibody dilution to retain a confluent cell monolayer (duplicate).

^*b*^ mAbs starting at 1:100 dilution were used for the MN assays.

We further evaluated the HA antigenicity of the four recombinant viruses using a ferret antiserum, a gold standard material for characterizing the HA antigenicity of influenza viruses (26), raised against an early A(H1N1)pdm09 isolate A/California/07/2009 (H1N1). In general, a tested virus showing more than an eightfold reduction in the ferret antiserum-based MN titer relative to a reference virus strain (20) is considered antigenically different from the reference strain (Table 3). The neutralization titers of the ferret antiserum against the CA04/HAΔ155G and CA04/HA-AB viruses were 8- to 16-fold lower than those against the WT CA04 and CA04/HA-ABins155G viruses, respectively. These results indicated that amino acid deletion at position 155 of H1 HA significantly altered HA antigenicity.

### Impact of the amino acid deletion on HA conformation

**Fig 7:**
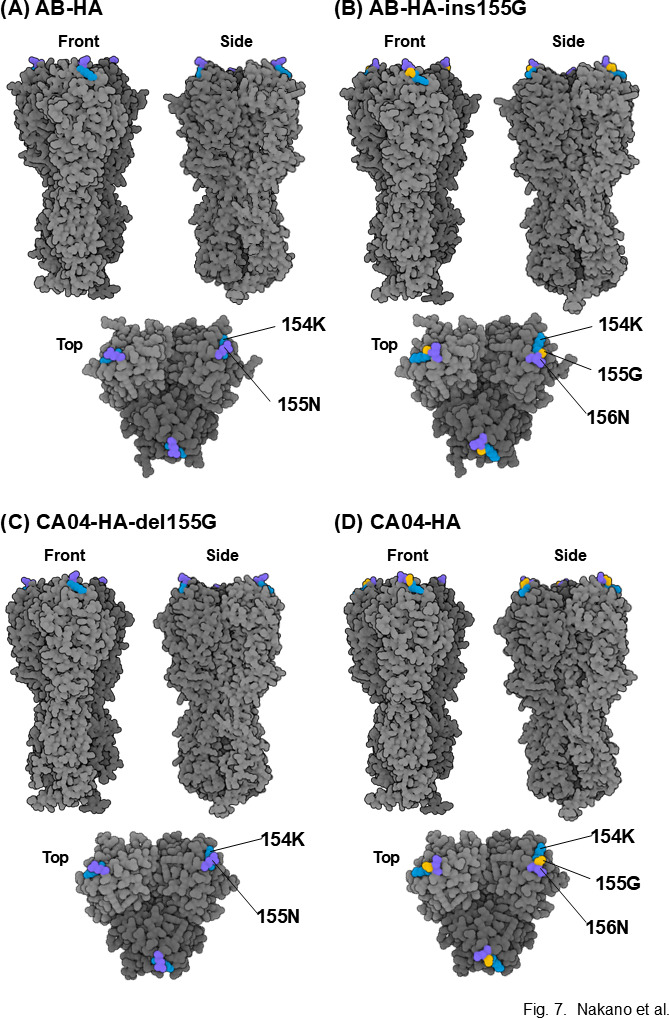
Predicted three-dimensional models of the H1 HA protein. The overall trimeric architecture generated by AlphaFold2 is shown. (**A**) HA from the wild-type AB strain. (**B**) AB strain-derived HA with an artificial G insertion at position 155. (**C**) CA04 strain-derived HA with an artificial G deletion at position 155. (**D**) HA from the WT CA04 strain. Residue 154 (lysine) is highlighted in blue, residue 155 (glycine) in yellow, and residue 156 (asparagine) in purple. For each panel, the front, side, and top views of the HA protein are displayed.

To assess the structural impact of the amino acid deletion at position 155 of H1 HA, we analyzed the HA amino acid sequences of the four recombinant viruses using the deep-learning-based protein structure prediction algorithm AlphaFold2-multimer (Fig. 7). The predicted three-dimensional models indicate that the deletion at position 155 does not cause significant conformational changes in the HA protein, suggesting that the amino acid deletion at position 155 composes linear, rather than conformational, epitopes of H1 HA.

## DISCUSSION

In this study, we genetically characterized 11 swIAV isolates from seven pig farms in Japan and demonstrated variations in the genetic constellation of swIAVs, even for the same subtype, circulating in the Japanese pig populations (Table 1). During genetic characterization, an amino acid deletion at position 155 of H1 HA was found in 1 of the 11 swIAV isolates, A/swine/Aichi/KU-AB/2018 (H1N2) (Fig. 5). Nucleotide sequence analysis of H1 HA genes from human, avian, and swine-origin influenza A viruses revealed that the amino acid at position 155 of H1 HA is rarely deleted. Artificial generation of recombinant influenza A viruses possessing the H1 HA gene with either an artificial insertion or deletion of G at position 155 (Fig. 5) allowed us to confirm the limited effect of amino acid deletion on viral replication (Fig. 6). In contrast, using these recombinant viruses, we demonstrated a greater impact of the amino acid deletion on HA antigenicity (Table 3), despite its limited effect on the predicted HA conformation (Fig. 7).

The neutralization titers of the mAbs and ferret antiserum against the four recombinant viruses generated in this study revealed that the amino acid deletion at position 155 of H1 HA significantly altered HA antigenicity (Table 3). The amino acid at position 155 is one of the constituent amino acids of the antigenic site Sa of H1 HA (21–23). One to two amino acid changes in the antigenic site Sa have been reported to dramatically affect the binding ability of human antibodies to the A(H1N1)pdm09 viruses (27). More specifically, substitution of the amino acid at position 155 of H1 HA causes antigenic drift (18, 19). However, the impact of amino acid deletion at this position on HA antigenicity remains unknown, partly because such HA deletion mutations have not been detected in human viruses. To the best of our knowledge, this is the first report demonstrating the great impact of the amino acid deletion at position 155 of H1 HA on HA antigenicity. Although the amino acid at position 155 of H1 HA is located within the Sa antigenic site, it is not part of the canonical receptor-binding pocket, which is primarily composed of the 130-loop, 190-helix, and 220-loop regions of HA (23). Sequence comparison and structural modeling indicated that these receptor-binding residues were conserved between the 155-deleted and 155G-carrying HAs analyzed in this study. Therefore, the amino acid deletion at position 155 is unlikely to directly alter receptor-binding specificity. However, we cannot exclude the possibility that this deletion may subtly affect receptor-binding properties through indirect or context-dependent mechanisms. Dedicated functional assays, such as glycan-binding analyses or infection studies in primary respiratory epithelial cells, will be required to fully evaluate the impact of this deletion on receptor preference.

The amino acid deletion at position 155 of H1 HA has rarely been reported (none in the case of humans and avians, and 0.17% [22 strains] in swine viruses). Nevertheless, our *in vitro* growth kinetics data for the four recombinant viruses in both AX4 and A549 cells indicated a limited effect of amino acid deletion on viral replication (Fig. 6). These results suggested that this minor amino acid deletion is capable of spontaneously emerging in pig populations without substantially compromising viral replicative capacity. It is to be noted that two of the 22 swine virus strains with the amino acid deletion were the AB and A/swine/Aichi/02/2016 (H1N2) strains, both isolated from pigs in Aichi Prefecture and sharing the highest nucleotide identity in their HA genes (Fig. 2). These repeated detections of HA-155-deletion viruses from pigs in the same region over at least two years indicate that swIAVs possessing an H1 HA gene segment with this deletion have been circulating locally rather than representing a single sporadic event. This information, along with the findings regarding HA antigenicity described above, suggests that such HA deletion mutations could, under appropriate conditions, become more common in larger pig populations.

Nine of the 11 isolates were categorized as the H1N2 subtype (Table 2), among which two isolates, A/swine/Chiba/KU-YH821-2/2021 (H1N2) and A/swine/Chiba/KU-YH821-4/2021 (H1N2), possessed HA and NA gene segments derived from the Japanese classical H1N2 swIAVs and from the human seasonal H3N2 influenza virus lineage, respectively (Table 2). While the NA gene segment from the AB strain was also derived from Japanese classical H1N2 swIAVs, its HA gene segment was derived from the A(H1N1)pdm09 virus lineage (Table 2). All HA and NA gene segments from the remaining seven H1N2 isolates were classified as Japanese classical H1N2 swIAVs (Table 2). Overall, we found that combinations in the HA and NA gene segments from our nine H1N2 swIAVs were divided into at least three types, although only 11 isolates were genetically characterized in this study. These results suggested variations in the genetic constellation of swIAVs circulating in Japanese pig populations, even for the same subtype.

In conclusion, we revealed the marked impact of the minor amino acid deletion at position 155 of H1 HA on HA antigenicity, despite its limited effect on viral replication in both AX4 and A549 cells. These results indicate that such deletion mutants can arise and be maintained in pig populations and may facilitate immune escape from pre-existing antibodies. However, we did not investigate replication in primary swine respiratory epithelial cells, receptor-binding preferences, or pathogenicity in animal models, nor did we assess neutralization by contemporary human sera. In this context, the fitness of these viruses in pigs remains to be determined. Our findings underscore the importance of continuous genetic and antigenic analysis of locally circulating swIAVs and of future studies in swine and human systems to evaluate the biological and public health implications of HA deletion mutations.

## MATERIALS AND METHODS

### Cells

AX4 cells, which are Madin-Darby canine kidney (MDCK) cells that overexpress human α2,6-sialyltransferase I and allow replication of human-origin influenza viruses more efficiently than wild-type MDCK cells (28), were maintained in minimum essential medium (MEM; Thermo Fisher Scientific, Waltham, MA) supplemented with 5% newborn calf serum and 2 μg/mL puromycin. Human lung epithelial A549 cells were maintained in Dulbecco’s modified Eagle’s medium (DMEM; FUJIFILM Wako Pure Chemical Corporation, Osaka, Japan) supplemented with 10% fetal calf serum. AX4 and A549 cells inoculated with either swine samples or swIAVs were cultured in MEM containing 0.3% bovine serum albumin and 2 μg/mL tolysulfonyl phenylalanyl chloromethyl ketone (TPCK)-treated trypsin. Human embryonic kidney-derived 293T cells were maintained in DMEM supplemented with 10% fetal calf serum. Plasmid-transfected 293T cells were cultured in Opti-MEM (Thermo Fisher Scientific). All the cell lines were maintained in a humidified incubator at 37°C and 5% CO_2_.

### Viruses

Eleven strains of influenza virus (Table 1) were isolated from samples collected from seven pig farms in Japan (Fig. 1). Lung tissue (farm A), oral fluid (farm B), and nasal swab (farms C–G) samples were collected from suckling (farms A–F) and weanling pigs (farm G) that showed influenza-like respiratory symptoms, such as cough and runny nose, and were subjected to swIAV isolation. Briefly, the swine samples were filtered through a 0.22 μm pore diameter membrane (Merck, Darmstadt, Germany), inoculated into AX4 cells, and cultured at 37°C for 2–3 days. The isolation of swIAV was confirmed by observation of the cytotoxic effect (CPE) in the inoculated AX4 cells, followed by detection of the influenza A viral M gene by real-time reverse transcription-PCR as previously described (29).

We artificially generated four recombinant influenza A viruses using reverse genetics (30) based on an early A(H1N1)pdm09 isolate A/California/04/2009 (H1N1; CA04 strain; see details below). These four viruses possessed the HA gene segment from A/swine/Aichi/KU-FKAB/2018 (H1N2; AB strain), designated as CA04/HA-AB virus, the AB strain-derived HA gene segment with an artificial glycine (G) insertion at position 155 (CA04/HA-ABins155G virus), the HA gene segment from the wild-type CA04 strain (WT CA04 virus), or the CA04 strain-derived HA gene segment with an artificial G deletion at position 155 (CA04/HAΔ155G virus). The remaining seven gene segments from these four recombinant viruses were derived from the CA04 strain.

These 11 swIAV isolates, as well as the four generated recombinant viruses, were propagated in AX4 cells, aliquoted, and stored at −80°C until further use. Stock virus titers were determined using 50% tissue culture infectious dose (TCID_50_) assays in AX4 cells. Briefly, 10-fold serial dilutions of the tested viruses were prepared in MEM containing 0.3% BSA and 2 μg/mL TPCK-treated trypsin and inoculated into AX4 cells in 96-well plates. The inoculated cells were cultured at 37°C for 3 days. The virus titer was calculated using the Reed-Muench method (31) based on the CPE in the inoculated cells as observed under a light microscope.

### Reverse genetics

The wild-type CA04 virus (Fig. 5) was generated using plasmid-based reverse genetics, as previously described (30). Briefly, 12 plasmids (eight of them to express individual gene segments from CA04 strain (20) and four plasmids to express a laboratory virus strain A/WSN/1933 [H1N1]-derived viral proteins PB2, PB1, PA, and NP that are required for transcription and replication of the influenza viral RNAs) were transfected into 293T cells cultured in Opti-MEM (Thermo Fisher) using the transfection reagent TransIT-293 (Mirus, Madison, WI) according to the manufacturer’s instructions. At 48 h post-transfection, supernatants were harvested and inoculated into AX4 cells for virus propagation. To generate a CA04 strain-based recombinant virus—the CA04/HAΔ155G virus (Fig. 5)—three consecutive nucleotides (guanine-guanine-adenine at nucleotide positions 463–465, encoding G at position 155 of HA) were deleted from a plasmid for the expression of the CA04 strain HA gene segment by site-directed mutagenesis.

We constructed a plasmid for the expression of the HA gene segment from the AB strain, as described previously (30). Using the constructed plasmid instead of the counterpart plasmid for the WT CA04 virus HA gene segment, we generated a CA04 strain-based HA variant named CA04/HA-AB virus (Fig. 5), using plasmid-based reverse genetics, as described above. To generate another CA04 strain-based recombinant virus, CA04/HA-ABins155G virus (Fig. 5), we inserted three consecutive nucleotides (encoding G at position 155 of HA) into the plasmid for expression of the AB strain HA gene segment constructed above.

### Sequencing

Viral RNA was extracted using the innuPREP Virus DNA/RNA kit (Analytik Jena, Thuringia, Germany) and reverse transcribed using SuperScript IV Reverse Transcriptase (Thermo Fisher Scientific, Waltham, MA) with universal primers specific for the 5′ end sequences conserved among influenza A viral gene segments (32). The viral genes were amplified by PCR using Tks Gflex DNA polymerase (Takara, Shiga, Japan) with viral gene segment-specific primer sets (32). The PCR products loaded on agarose gels were purified using the Wizard SV Gel and PCR Clean-Up System (Promega Corporation, Madison, WI). The DNA sequences of the PCR products were determined by Sanger sequencing at FASMAC Co., Ltd. (Atsugi, Japan).

### Phylogenetic analysis

The viral gene sequences from the 11 swIAV isolates determined in this study were phylogenetically analyzed using Japanese swIAVs and human seasonal influenza vaccine strains retrieved from the GISAID database (https://gisaid.org/) in September 2022. Nucleotide sequences were aligned using the MAFFT software version 7.397 (https://mafft.cbrc.jp/alignment/software/). Phylogenetic trees of HA and neuraminidase (NA) gene segments were constructed using the maximum likelihood method with 1,000 bootstrap replicates in MEGA version 7 (33).

### Analysis of amino acid variation at position 155 of H1 HA

Nucleotide sequence data for the H1 HA gene segment from influenza A viruses of human (64,146 strains), avian (1,017 strains), and swine (12,801 strains) origin were retrieved from the GISAID database in September 2022. The retrieved nucleotide sequences were aligned using MAFFT software version 7.397. Based on the deduced amino acid sequences, the variation in the amino acids at position 155 of HA was analyzed.

### Growth kinetics of recombinant viruses

AX4 and A549 cells were inoculated with the WT CA04, CA04/HAΔ155G, CA04/HA-AB, and CA04/HA-ABins155G viruses in six-well plates, at a multiplicity of infection of 0.001 in triplicates. After virus adsorption at 37°C for 1 h, the inoculants were replaced with MEM-0.3% BSA containing 2 μg/mL of TPCK-treated trypsin. Virus titers in supernatants collected at 12, 24, 48, and 72 h post-inoculation were determined by TCID_50_ assays in AX4 cells, as described above.

### Antibodies

Ten clones of mouse monoclonal antibodies (mAbs) that recognize G at position 155 of H1 HA from A/Narita/1/2009 (H1N1), the first A(H1N1)pdm09 isolate in Japan, that is, mAb clones N73, N230, N327, N329, N334, N343, N345, N408, 3N38, and 3N64 (25), were kindly provided by Dr. Ayato Takada from the Research Center for Zoonosis Control, Hokkaido University. Ferret antiserum raised against A/California/07/2009 (H1N1; an initial A[H1N1]pdm09 vaccine strain) (34) was kindly provided by Dr. Shinji Watanabe at the Influenza Virus Research Center of the National Institute of Infectious Diseases. It was previously demonstrated that the CA04 strain, A/Narita/1/2009 (H1N1), and A/California/07/2009 (H1N1) are indistinguishable in their HA antigenicity (18).

### Microneutralization assay

Neutralization titers of the mAbs and the ferret antiserum against the viruses tested were determined by microneutralization (MN) assays, as described previously (35). Briefly, two-fold serial dilutions of each mAb were mixed with 100 TCID_50_ of each virus in MEM-0.3% BSA and incubated at 37°C for 1 h. The virus-mAb mixtures were inoculated into AX4 cells in 96-well plates in duplicates and cultured for three3 days without removing the inoculant. Ferret antiserum was treated with a receptor-disrupting enzyme (RDE II, Denka Seiken Co., Ltd., Tokyo, Japan). Two-fold serial dilutions of ferret antiserum were mixed with 100 TCID_50_ of each virus tested in MEM-0.3% BSA and incubated at 37°C for 1 h. The virus-antiserum mixtures were inoculated into AX4 cells in 96-well plates in duplicates and further incubated at 37°C for 1 h. After removing the inoculant, the inoculated cells were washed twice with PBS and cultured for 3 days. The neutralization titer was determined based on CPE in the inoculated cells, as observed under a light microscope.

### HA conformation prediction

Three-dimensional models of the HA proteins from WT CA04, CA04/HAΔ155G, CA04/HA-AB, and CA04/HA-ABins155G viruses were predicted using the deep-learning-based protein structure prediction algorithm AlphaFold2. Briefly, the HA amino acid sequences of each virus were individually submitted to the web-based AlphaFold2 server (https://cryonet.ai/af2/) for structural prediction. The resulting models were then subjected to visual assessment to evaluate the potential structural impact of the amino acid deletion at position 155 in H1 HA.

## Data Availability

Nucleotide sequence data generated in this study are publicly available in the GISAID database. The remaining data supporting the findings of this study are available from the corresponding author upon reasonable request.
